# Comparison of sublingual therapeutic vaccine with antibiotics for the prophylaxis of recurrent urinary tract infections

**DOI:** 10.3389/fcimb.2015.00050

**Published:** 2015-06-03

**Authors:** María F. Lorenzo-Gómez, Bárbara Padilla-Fernández, María B. García-Cenador, Álvaro J. Virseda-Rodríguez, Isidoro Martín-García, Alfonso Sánchez-Escudero, Manuel J. Vicente-Arroyo, José A. Mirón-Canelo

**Affiliations:** ^1^IBSAL Salamanca Biomedical Research InstituteSalamanca, Spain; ^2^Department of Surgery, University of SalamancaSalamanca, Spain; ^3^Department of Urology, University Health Complex of SalamancaSalamanca, Spain; ^4^Department of Urology, University Hospital of the Canary Islands' ComplexTenerife, Spain; ^5^Primary Care Centre “Universidad Centro”Salamanca, Spain; ^6^Primary Care Centre “Peñaranda”Salamanca, Spain; ^7^Primary Care Centre “Capuchinos”Salamanca, Spain; ^8^Department of Preventive Medicine and Public Health, University of SalamancaSalamanca, Spain

**Keywords:** recurrent urinary infections, bacterial vaccine, Uromune (MV 140)

## Abstract

**Objective:** To compare the clinical impact of a prophylactic treatment with sublingual immunostimulation in the prevention of recurrent urinary tract infections (rUTIs) with the use of antibiotics.

**Material and Methods:** Retrospective cohort study evaluating the medical records of 669 women with rUTIs; 339 had a 6-month prophylaxis with antibiotics and 360 a 3-month prophylaxis with a sublingual bacterial preparation (MV 140-Uromune®). The time frame after the prophylaxis-period until the appearance of a new infection (assessed by uroculture) was scored and followed during 1 year. The absolute risk reduction (ARR) and number needed to treat (NNT) were also calculated.

**Results:** All patients treated with antibiotics experienced a new UTI during the scoring period of 12 months, being 19 days the median number of days free of UTIs (range 5–300). In the group treated with the bacterial preparation, 35 (9.7%) patients experienced an UTI in the same period. Kaplan-Meier curves comparing the accumulated survival (disease-free time) between both groups were significant different (*P* < 0.0001). The absolute risk reduction (ARR) was 90.28% (87.18–93.38) and the number needed to treat (NNT) 1.1 (1.1–1.1).

**Conclusions:** These results suggest that the treatment with this bacterial preparation significantly reduces the incidence of rUTIs, arising as an effective strategy to reduce the frequency of rUTIs. It reduces antibiotic consumption, matching the current recommendations due to the raise of antimicrobial resistance. Randomized, double-blind and placebo-controlled, clinical trials are needed to establish, more accurately, the clinical impact of this bacterial preparation in patients with rUTIs.

## Introduction

Symptomatic urinary tract infections (UTIs) are defined when there are clinical symptoms indicative of infection and the presence of pathogens can be verified (Johansen et al., 2011). These are the most frequent bacterial infections in human (Foxman, 2002; Nicolle, 2005) and the first infection recorded in hospital the setting (Salvatore et al., 2011), in which 2% of the hospitalized patients acquire UTIs. In the 1980s decade, nosocomial infections accounted for more than 500,000 cases per year (Mayer, 1980; Turck and Stamm, 1981), being the bladder the most common site of infection (cystitis). At least 25% of the patients having an UTI will have a recurrence within 6 months, with 48% of them happening during the 1 year (Salvatore et al., 2011). Twenty two percent will have recurrent urinary tract infections (rUTIs). Because the vast majority of these infections are of their bacterial origin (Foxman, 2002; Nicolle, 2005), antibiotics are the main etiological treatment.

rUTIs have an important clinical impact on the health and quality of life of patients, together with a great economic impact. The annual cost in the United States is estimated to be more than 2.5 billion dollars (Rahn, 2008), including millions of courses of therapy with antibiotics (Foxman et al., 2000). Each episode of acute UTI in pre-menopausal women is associated with 6.1 days of disability, 2.4 days of school or work absenteeism and, in average, 0.4 days in bed (Foxman, 2002).

Women are 8–30 times more likely to have UTIs than men (Cox et al., 1968; Naber et al., 2009), with a peak between 16 and 35 years of age. Usually, over one third of women report at least one UTI in their lifetime (Salvatore et al., 2011), becoming a common condition diagnosed and treated by general practitioners, urologists, gynecologists and other health care providers (Foxman, 2002). From the epidemiological point of view, 2 to 3% of women between 15 and 24 years of age have bacteriuria, raising to 20% in women between 65 and 80 years and to 25–50% in women older than 80 years (Mulholland, 1986). The European Commission estimates that the population group over 65 years of age will increase 1.5-times in Europe between 2000 and 2030 (European_Commission, 2010), and the United States Census Bureau estimates that number of women older than 65 will double in the United States (U.S._Census_Bureau, 2008). Therefore, this situation clearly anticipates that the number of UTIs will significantly increase in the upcoming years.

The current advised therapy for the prevention of rUTIs is the continuous prophylaxis with antibiotics, being the most recommended option the treatment with Sulfamethoxazole/Trimethoprim (SMX/TMP) or Nitrofurantoin for a period of 6 months (Nicolle and Ronald, 1987; Hooton, 2001; Grabe et al., 2013). However, long term antibiotic consumption is not innocuous for the patient, and problems derived from the deleterious effects on the gut microbiota and/or the potential adverse events associated with its use is always a concern. In addition, the continuous use of antibiotics is associated with the widely increase of antimicrobial resistance to antibiotics creating a dramatic situation that demands a global challenge (Howard et al., 2014) involving governments (Walsh, 2014), health (European_Medicines_Agency, 2014; Food_and_Drug_Administration U. S., 2014; World-Health_Organization, 2014), and economic (World-Economic-Forum, 2014) organizations, among others, leading to the conclusion that the use of antibiotics as suppressive therapy or long-term prophylaxis may no longer be advisable (Pallett and Hand, 2010).

We previously found in an observational retrospective study when treating rUTIs, that the improvement of the patients treated with this sublingual preparation (for a period of 3 months) compared with the prophylactic treatment with antibiotics (for a period of 6 months) was 75% in the first 3 months and 86 and 77% at 9 and 15 months (Lorenzo-Gómez et al., 2013). To investigate the preventative value of both treatments, this study aims to address specifically the disease-free time after each treatment course evaluating the time between the onset of a new infection after the prophylactic course of a commercially available mucosal immunostimulant (MV-140 Uromune®) compared with the currently recommended prophylaxis with SMX/TMP or nitrofurantoin.

## Methods

### Study design

**Figure 1:**
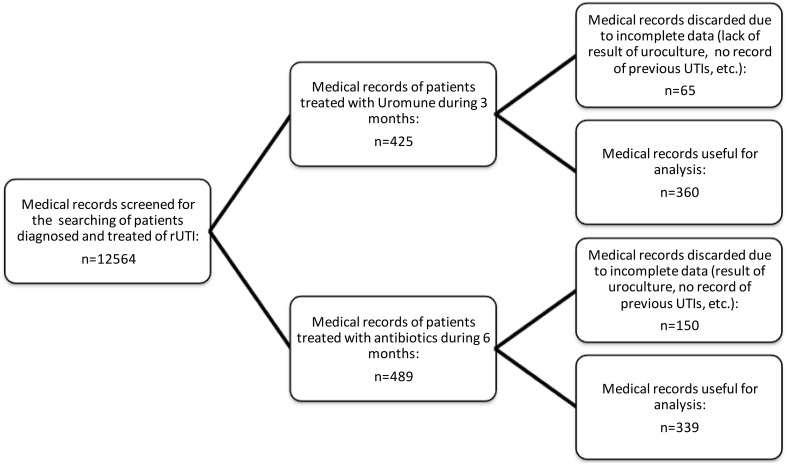
**Flow chart of the study**.

It was a cohort to estimate the absolute risks in subjects with rUTIs, in order to determine whether the prophylactic treatment with Uromune is associated with a lower risk of new UTI. Recurrent UTI was defined as 3 or more culture-documented infections in a year, or 2 or more in 6 months (Rahn, 2008). The Figure 1 shows the flow-chart and distribution of the subjects.

The study protocol was reviewed and approved by the Ethical Review Board of the University of Salamanca (Spain).

### Study population

Sample size was calculated using the software Epidat 3.1 (Xunta de Galicia and PanAmerican Health Organization). The sample size estimation for survival analysis is based on the publication by Ahnn (Ahnn and Anderson, 1995). For 2 groups, with a confidence level of 95%, power of 80% and expecting a probability of survival of 40% in the groups treated with Uromune® and 30% in the group treated with prophylaxis with antibiotics, the estimated number of patients per group is a minimum of 324.

First, we screened 12564 medical records belonging to 5 primary care centers and the Urology Unit of the University Hospital of the health area of Salamanca (Spain), searching for those in which was clearly stated that the patients were diagnosed and treated of rUTI, finding that 425 were treated with Uromune and 489 with SMX/TMP or Nitrofurantoin. All definitions of “rUTI” found in the files studied were accepted except 65 in the group of patients treated with Uromune and 150 in the group of antibiotics. These were discarded due to the lack of relevant data (no information regarding urocultures, number of infections before the initiation of treatment, etc.). It was checked that the treating doctors followed local and international guidelines (Grabe et al., 2010, 2013) regarding diagnosis (urinary irritative symptomatology -dysuria, frequency and urgency- with the absence of vaginal discharge or irritation and the corresponding positive uroculture of >10^3^ cfu/mL of uropathogens in a mid-stream sample of urine in acute uncomplicated cystitis in women).

Finally, we reviewed the data from the medical records that had enough information regarding the description of the infection, the result of the uroculture, data of antibiogram and antibiotic resistance, etc. The medical records were of 360 women who were treated with Uromune® for a period of 3 months (group A) and the data of 339 who were treated with SMX/TMP (*n* = 271), or Nitrofurantoin (*n* = 68) for a period of 6 months (Group B) (Figure 1). Nitrofurantoin was chosen when the infective bacteria were resistant to SMX/TMP (57 *Escherichia coli*, 10 *Proteus mirabilis* and 1 *Klebsiella pneumoniae*). Epidemiological data related to age, years of evolution of rUTIs, medical and surgical background, sexual habits, obstetric/gynecological records, specific treatment and evolution were recorded and analyzed (**Table 2**). Patients with chronic kidney insufficiency and/or under treatment with immunosuppressors were not included. There were no differences between groups, regarding age, ethnicity, dietetic habits, coital activity, hormonal status, or usual treatment. The bacteria-positive urocultures responsible of the UTIs in the 3 months previous to the prophylactic treatment are shown in **Table 3**.

### Treatments

Patients in group A received Uromune® for a period of 3 months. This is a sublingual bacterial immunostimulant produced under GMPs (as specific named patient formulation) by Inmunotek (Madrid, Spain) and marketed in Spain by Q-Pharma (Alicante, Spain). The preparation consisted of 2 vials containing a suspension of 10^9^ inactivated whole bacteria/mL, containing a mixture of equal amounts of selected strains of *Escherichia coli, Klebsiella pneumoniae, Proteus vulgaris*, and *Enterococcus faecalis*. These microorganisms are those producing the majority of rUTIs in Europe (Andreu and Planells, 2008). The preparation was delivered by means of a pump-spray to the oral/sublingual mucosa and the dose was 2 puffs of 100 μL each (10^8^ bacteria/puff) daily, avoiding the concomitant intake of food or beverage. The delivered dose was maintained under the tongue for a period of 1–2 min and then swallowed.

Patients in group B received a daily dose of SMX/TMP (200/40 mg/day) or nitrofurantoin (100 mg/day) orally as a prophylactic treatment for a period of 6 months (Nicolle and Ronald, 1987; Hooton, 2001; Grabe et al., 2013).

### Evaluation

The following data was collected from the medical records: (1) the number of years that patients had rUTIs before initiating prophylaxis; (2) number of UTIs with the corresponding urocultures (UC) in the previous 3 months and causative bacteria with the corresponding antibiogram; (3) the elapsed time from the last day of the prophylactic treatment since the first episode of UTI confirmed by UC and the identification of the causative bacteria, establishing as limit of review 12 months after the end of the prophylactic treatment. The time period before the onset of a new UTI after the prophylactic treatment was the main outcome of this study.

### Statistics

The Excel spreadsheet (Microsoft, Inc. USA) with the statistical add-in XLStat (Addinsoft, Paris, France) was used. The results were analyzed for normality (Shapiro-Wilk), showing that all the outcomes did not follow a normal distribution. Descriptive statistics were expressed as the median with the first and third interquartile range (IQR).

Mann-Whitney test and Fisher's exact test were used to compare between groups the epidemiological data (Table 1) and the bacteria responsible (Table 2). The proportions of patients in each treatment group remaining infection-free over time were compared using Kaplan-Meier's estimator. Absolute risk reduction (ARR) and number needed to treat (NNT), with the corresponding 95% confidence intervals, were also calculated.

**Table 1:** **Chart of the study**.

	**Free of new UTI after the prophylactic treatment**	
	Yes	No
Patients treated 3 months with Uromune	325	35
Patients treated 6 monts with antibiotics^*^	0	339

* *SMX/TMP or Nitrofurantoin*.

**Table 2:** **Demographic data of the patients**.

	**A (*n* = 360)**	**B = 339**	***P***
Age^#^	60 (44–70)	59 (49–69)	0.3384
Age range	17–85	19–91	
Years of evolution before prohylactic treatment^#^	6 (4–8)	7 (4–9)	0.7523
Clinical variables^*^			
Regular sexual activity	276	270	0.3609
Menopause	170	136	0.7572
Miltiparous	163	139	0.2849
Nulliparous	91	87	0.9309
Diabetes mellitus	66	51	0.2655
Drug allergy	66	68	0.5658
Arterial hypertension	118	92	0.1168
Eutocic childbirth	197	191	0.7035
Dystocic childbirth	75	59	0.2903
Breathing disorders	66	47	0.1232
Stomach disorders	102	83	0.2653
Surgical correction of urinary incontinence	70	81	0.1681
Surgical correction of cystocele	34	38	0.4576
Hysterectomy	127	106	0.2967
Double oophorectomy	111	89	0.2091
Smoking habit	113	91	0.2118
Obesity	102	75	0.0676
Antidepresant/anxiolytic drugs	138	123	0.5848

* Fisher's exact test;

# *Mann-Whitney's test*.

## Results

Both groups of patients were similar in the number of UTIs, UC+ (Table 2) and antibiotic resistances (Table 3) before the commencement of the prophylactic treatments.

**Table 3:** **UTIs with the corresponding positive urocultures and bacteria responsible in the 3 months previous to the initiation of the prophyactic treatment**.

	**A (*n* = 360)**	**%**	**B (*n* = 339)**	**%**	***P***
Positive urocultures^*^	515	100	503	100	
*Citrobacter farmeri*	0	0	0	0	>0.9999
*Citrobacter freundii*	4	1	0	0	0.1244
*Corynebacterium sp*	0	0	4	1	0.0592
*Citrobacter koseri*	4	1	0	0	0.1244
*Enterobacter aerogenes*	4	1	6	1	0.5424
*Enterobacter cloacae*	11	2	0	0	0.0009
*Enterococcus avium*	0	0	3	1	0.1203
*Enterococcus faecium*	0	0	4	1	0.0592
*Enterococcus faecalis*	42	8	18	4	0.0028
*Escherichia coli*	310	60	333	66	0.0513
*Klebsiella oxytoca*	21	4	12	2	0.1569
*Klebsiella pneumoniae*	35	7	54	11	0.0544
*Morganella morganii*	4	1	4	1	>0.9999
*Proteus mirabilis*	32	6	39	8	0.4629
*Proteus vulgaris*	0	0	0	0	>0.9999
*Pseudomonas aeruginosa*	4	1	0	0	0.1244
*Routella planticola*	8	2	0	0	0.0076
*Salmonella*	4	1	0	0	0.1244
*Staphylococcus aureus*	0	0	0	0	>0.9999
*Staphylococcus saprophyticus*	16	3	5	1	0.0253
*Streptococcus agalactiae*	16	3	21	4	0.4047

* *Comparison: Fisher's extact test*.

Patients of groups A had a median of 6 (Cox et al., 1968; Mayer, 1980; Turck and Stamm, 1981; Foxman et al., 2000; Rahn, 2008; Salvatore et al., 2011) UTIs in the previous 12 months before to initiate the prophylactic treatment. The figures in group B were 6 (Salvatore et al., 2011; Mayer, 1980; Turck and Stamm, 1981; Rahn, 2008; Foxman et al., 2000; Cox et al., 1968) (*P* = 0.7521).

### Safety

No report of side effect, either local, in the oral mucosa, or systemic, was recorded after the use of MV-140 Uromune®.

### Urinary tract infections

**Figure 2:**
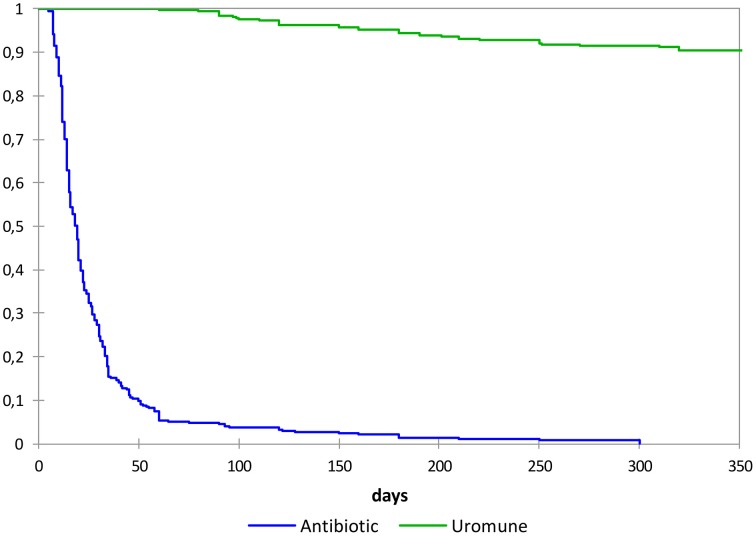
**Evolution of percent of women remaining free of UTI**.

During the period of time of the prophylactic treatment, 290 patients (81%) of group A were free of infection in contrast with 9 (3%) of group B (*P* < 0.0001). After this treatment, in all group A, 35 (9.7%) patients experienced a new UTI episode, being the median of 180 (105–325) days (range 60–360) after ending the treatment with the bacterial preparation. In contrast, all patients in group B experienced the first new UTI episode in the 12 months review. The median time to experience a new UTI was 19 (12–30) days, range 5–300 days (Tables 1, 4). There was no difference between the patients treated with SMX/TMP and those treated with nitrofurantoin (*P* = 0.33). The differences between both antibiotic subgroups in the accumulated survival (Kaplan-Meier, Figure 2) were not significant (log rank *P* value = 0.75). The differences between groups A and B in the accumulated survival (Kaplan-Meier) were highly significant (log rank *P* value < 0.0001). The ARR was 90.28% (87.18–93.38) and NNT 1.1 (1.1–1.1).

**Table 4:** **UTIs with the corresponding positive urocultures and bacteria responsible after the period of the the prophyactic treatment**.

	**A (*n* = 360)**	**%**	**B (*n* = 339)**	**%**	***P***
Patients with UTI^*^	35		339		<0.0001
Median of days of evolution until first UTI^#^	180 (105–235)		19 (12–30)		<0.0001
Positive urocultures^*^	35	100	339	100	<0.0001
*Citrobacter farmeri*	0	0	4	1	>0.9999
*Citrobacter freundii*	0	0	4	1	>0.9999
*Corynebacterium sp*	0	0	0	0	>0.9999
*Citrobacter koseri*	0	0	0	0	>0.9999
*Enterobacter aerogenes*	0	0	5	1	>0.9999
*Enterobacter cloacae*	0	0	0	0	>0.9999
*Enterococcus avium*	0	0	0	0	>0.9999
*Enterococcus faecium*	0	0	4	1	>0.9999
*Enterococcus faecalis*	4	11	15	4	0.0901
*Escherichia coli*	25	71	220	65	0.5756
*Klebsiella oxytoca*	3	9	5	1	0.0304
*Klebsiella pneumoniae*	0	0	39	12	0.0365
*Morganella morganii*	0	0	0	0	>0.9999
*Proteus mirabilis*	0	0	24	7	0.1487
*Proteus vulgaris*	0	0	3	1	>0.9999
*Pseudomonas aeruginosa*	0	0	0	0	>0.9999
*Routella planticola*	0	0	0	0	>0.9999
*Salmonella*	0	0	0	0	>0.9999
*Staphylococcus aureus*	3	9	0	0	0.0008
*Staphylococcus saprophyticus*	0	0	4	1	>0.9999
*Streptococcus agalactiae*	0	0	12	4	0.6134

# *only patients who experienced UTI. Comparison: Mann-Whitney's test*.

* *Comparison: Fisher's extact test*.

Regarding antimicrobial resistances following prophylaxis, the total number of resistant bacteria decreased in group A because the decrease in new UTIs. However, the statistical analysis showed that there were no differences between groups in the increase of antibiotic-resistant bacteria after the prophylactic treatment (Table 5).

**Table 5:** **Number of bacteria producing infections and number of bacteria resistant to different antibiotics, before and after the prohylactic treatment**.

	**Group A**		**Group B**		P^*^	
	**Isolated bacteria**	**Resistant**	**Isolated bacteria**	**Resistant**	
Before prophylaxis	515	199 (39%)	503	219 (44%)	0.1262
After prophylaxis	35	11 (31%)	339	153 (45%)	0.1522
P^*^		0.4738		0.6714	

* *Fisher's exact test*.

*No differences were observed intra and inter groups*.

Some patients in group A had previous infections with bacteria that were not included in the MV-140 formulation (Table 3). After the 3 months of treatment with this preparation, the patients remained UTI-free 12 months later (Table 4), and therefore, also free of infections due to these bacteria.

## Discussion

In this retrospective study we have compared the administration of a sublingual bacterial preparation (MV-140 Uromune®) with the currently recommended use of antibiotics for a period of 6 months (Grabe et al., 2013).

In this study, patients treated for a period of 3 months with MV-140 Uromune® had a significantly longer UTI-free time interval than patients treated during 6 months with SMX/TMP or nitrofurantoin. The review of the medical records indicated that 90.3% (325 patients) of the patients treated with MV-140 Uromune® remained free of new UTI, in contrast with 0% of patients treated with the conventional antibiotic prophylaxis. Thus, patients treated with MV-140 Uromune® experienced a much better clinical improvement than those treated with antibiotics. The ARR of 93.38 and the NNT of 1.1, as measures of effectiveness of treatment specificity, means that the favorable outcome occurs practically in every patient who received MV-140 Uromune® and in no patient receiving antibiotic.

To the best of our knowledge, this is the first study to report a survival analysis of 12 months after the use of mucosal sublingual immunostimulation showing such a degree of effectiveness. Hopkins (Hopkins et al., 2007) reported a survival analysis for a period of 160 days using mucosal vaginal immunostimulation vs. placebo. In this study each patient received 3 initial vaginal suppositories weekly followed by 3 additional monthly suppositories. No significant differences were observed, except in a group of patients who received an additional booster. The patients free of new UTI in the 160 days of follow-up were 16.7% in the placebo group, 25% in the active group and 46% in the active group with a booster. In our study, 340 (94.4%) patients who received sublingual prophylaxis were free of a new UTI after 160 days, demonstrating a higher clinical benefit.

The results obtained in the current study reinforce those shown in a previous publication (Lorenzo-Gómez et al., 2013), in which the main outcome was the number of UTIs after the initiation of the prophylactic treatment with MV-140 Uromune®, or antibiotics. Patients treated with MV-140 Uromune® for a 3-month period had an improvement greater than 75% in the number of new UTIs when compared to patients treated with SMX/TMP for a period of 6 months. The benefit of Uromune® was maintained after an observation period of 9 and 15 months (86 and 77% of improvement, respectively).

The use of bacterial preparations to prevent rUTIs was recommended in 2009 by the European Association of Urology (Grabe et al., 2010) and in 2013 (Grabe et al., 2013) recommended one preparation and the conduction of large phase III studies for other immunotherapeutics. The comparison of an oral bacterial lysate of 10^9^ *E. coli* with nitrofurantoin, as a prophylaxis for rUTIs in girls, was reported by Lettgen et al (Lettgen, 1996), showing that the efficacy of the long-term administration of this bacterial lysate was comparable to that of nitrofurantoin.

Bauer reported in 2005 (Bauer et al., 2005) a double blind, placebo controlled study in 454 women using a similar approach consisting in an oral administration of capsules containing freeze-dried lysate of *E. coli*. Patients were treated with 1 capsule (active or placebo) per day for 90 days, followed by 3 months without treatment, and then the capsules were taken again only the first 10 days in months 7, 8, and 9. These patients were followed up during 12 months, reporting a 34% reduction of UTIs in patients treated with the bacterial lysate when compared to placebo. The same authors (Bauer et al., 2002) reported in 2002 a meta-analysis performed on 5 studies of this oral bacterial lysate of 10^9^ *E. coli* compared with placebo in double-blind studies in patients with UTIs (601 women), showing a superiority of 35% of this treatment over placebo. The drug was well tolerated and patients' compliance was excellent in all studies. However, none of these studies reported the time free of new UTIs after the prophylactic treatment.

In the present study, the prophylactic benefit of sublingual immunostimulant was greater than the described with the current available oral bacterial lysates (Lettgen, 1996; Bauer et al., 2002, 2005) or vaginal whole bacteria preparations (Hopkins et al., 2007). These differences could be explained by the form by which the bacteria are formulated (whole inactivated bacteria vs. lysate) (Sato et al., 2000; Underhill and Ozinsky, 2002; Blander and Sander, 2012; Rosadini and Kagan, 2015), the inductive mucosal site (Quiding et al., 1991; Kozlowski et al., 1997; Eriksson et al., 1998; Holmgren and Czerkinsky, 2005; Çuburu et al., 2007) (gut or vaginal vs. sublingual/oral mucosa) and/or the bacterial strains used (Yu et al., 2007; Wiles et al., 2008; Croxen and Finlay, 2010; Ulett et al., 2013). Sublingual mucosa is an inductive site for generating broad spectrum mucosal and systemic immune responses, including the respiratory and genitourinary tracts (Holmgren and Czerkinsky, 2005; Çuburu et al., 2007), with a high degree of efficacy and persistence of the immune response (Negri et al., 2010). Sublingual immunostimulation induces systemic humoral dose-dependent immune responses (Çuburu et al., 2007), mucosal antibody responses (Çuburu et al., 2007) and an immune stimulating effect on CD4+ T helper cell responses to bacteria (Alecsandru et al., 2011).

An interesting finding in the current study is that patients of group A who had previous infections with bacteria not included in MV-140 Uromune®, such as *Enterobacter aerogenes, Enterobacter cloacae, Morganella morganii, Pseudomonas aeruginosa, Routella planticola, Salmonella, Staphylococcus saprophyticus*, and *Streptococcus agalactiae* did not have new infections with these bacteria, suggesting a broad immune stimulation. This is supported by the enhancement of T cell responses to flu antigens in patients treated with other sublingual bacterial preparations (Alecsandru et al., 2011).

In summary, the results obtained in this study favor the use of bacterial immunostimulants instead of antibiotics for the prophylactic treatment of rUTIs as a reasonable strategy to avoid the latter in a safe and effective way. This approach is in line with the recommendations of all the social agents, promoting new treatment alternatives against bacterial diseases of high prevalence and those that may precipitate secondary bacterial diseases (World_Health_Organization, 2001; Centers_for_Disease_Control_and_Prevention, 2011; European_Medicines_Agency, 2011; Food_and-Drug_Administratrion, 2011).

We acknowledge that because this study is a compilation of existing data with the only criteria of having rUTIs under prophylaxis with Uromune or with antibiotics, it doesn't provide deeper and more accurate outcomes as those that can be obtained in a prospective, explanatory controlled trial conducted in clinically experimental conditions. Patients included in this study are those that exist records regarding follow-up. A possible weakness is the vague definition of UTI in the medical records and a possible source of bias was that we didn't include patients that received treatment but there was not follow-up. Nevertheless, the sample size and the data collected from these medical records provides clinically valuable information of the patients treated under “real world” conditions, not only regarding the effectiveness of Uromune, but in the low degree of clinical benefit of SMX/TMP or Nitrofurantoin as prophylactic treatment. We do believe, however, that further prospective double-blind, placebo-controlled, randomized clinical trials are needed to establish more accurately the clinical impact of this bacterial preparation in patients with rUTIs.

The results obtained in this study show that treatment with the bacterial preparation reduces rUTIs effectively, far beyond than with the recommended antibiotic prophylaxis. Therefore, this approach arises as an effective strategy to reduce the frequency of rUTIs. Moreover, it reduces antibiotic consumption, which is in line with the current recommendations of Governments, Health and Regulatory Authorities due to the raise of antimicrobial resistances.

### Conflict of interest statement

The authors declare that the research was conducted in the absence of any commercial or financial relationships that could be construed as a potential conflict of interest.
